# Supplementary figures and images for: Uncovering the Core Microbiome and Distribution of Palmerolide in Synoicum adareanum Across the Anvers Island Archipelago, Antarctica
Source: Mar Drugs. 2020 Jun 2;18(6):298. doi: 10.3390/md18060298 (PMC7345734; doi:10.3390/md18060298)

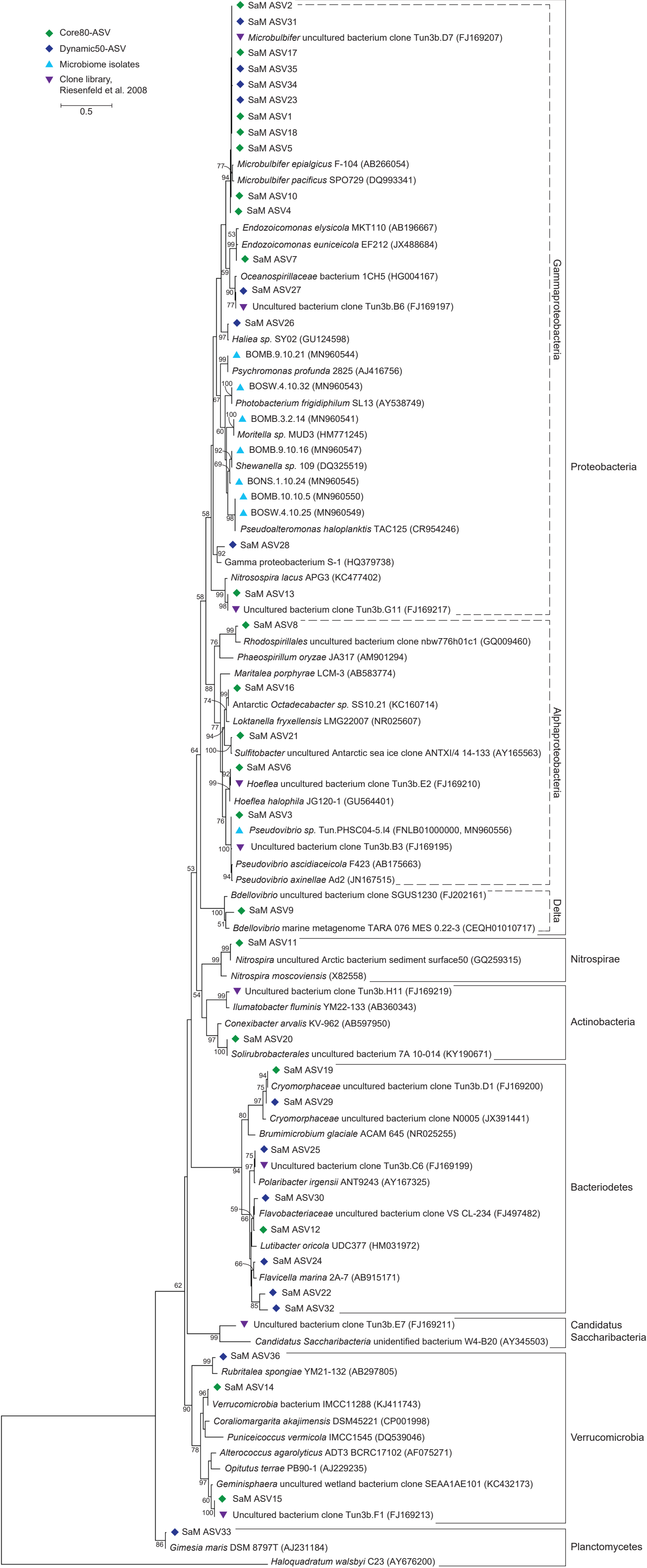

Supplement: Supplementary file 1 [file marinedrugs-18-00298-s001.zip › FigS2PhylogeneticTree_v3.pdf]
